# Context-independent encoding of passive and active self-motion in vestibular afferent fibers during locomotion in primates

**DOI:** 10.1038/s41467-021-27753-z

**Published:** 2022-01-10

**Authors:** Isabelle Mackrous, Jérome Carriot, Kathleen E. Cullen

**Affiliations:** 1 Department of Physiology, McGill University, Montreal, QC Canada; 2 Department of Biomedical Engineering, Johns Hopkins University, Baltimore, MD USA; 3 Department of Otolaryngology-Head and Neck Surgery, Johns Hopkins University School of Medicine, Baltimore, USA; 4 Department of Neuroscience, Johns Hopkins University School of Medicine, Baltimore, USA; 5 Kavli Neuroscience Discovery Institute, Johns Hopkins University, Baltimore, USA

**Keywords:** Neural encoding, Sensory processing

## Abstract

The vestibular system detects head motion to coordinate vital reflexes and provide our sense of balance and spatial orientation. A long-standing hypothesis has been that projections from the central vestibular system back to the vestibular sensory organs (i.e., the efferent vestibular system) mediate adaptive sensory coding during voluntary locomotion. However, direct proof for this idea has been lacking. Here we recorded from individual semicircular canal and otolith afferents during walking and running in monkeys. Using a combination of mathematical modeling and nonlinear analysis, we show that afferent encoding is actually identical across passive and active conditions, irrespective of context. Thus, taken together our results are instead consistent with the view that the vestibular periphery relays robust information to the brain during primate locomotion, suggesting that context-dependent modulation instead occurs centrally to ensure that coding is consistent with behavioral goals during locomotion.

## Abstract (web summary)

Using experimental and computational approaches the authors show that the vestibular efferent system does not modulate peripheral coding during locomotion. Instead, vestibular afferents unambiguously convey information in a context independent manner.

## Introduction

The vestibular system computes precise estimates of our self-motion relative to the world and our orientation relative to gravity to ensure accurate perception and motor control. The receptor cells of the vestibular sensory organs detect head motion, and in turn, ascending projections from the afferent fibers of the VIII nerve transmit this information to the vestibular nuclei and cerebellum (reviewed in ref. ^1^). Importantly, the brain also sends descending connections from the central nervous system back to peripheral vestibular organs (i.e., the efferent vestibular system (EVS)). To date, however, the functional role of the EVS has remained the focus of scientific debate. In mammals, the EVS comprises ~300 cell bodies in the brainstem, which send bilateral projections back out to the vestibular hair cells and afferent nerve fibers of the vestibular organs^2–5^. While the responses of afferents to artificial stimulation of the EVS have been described in mammals^6–9^, the question of whether and how the EVS is actually activated during everyday life remains unanswered.

A long-standing hypothesis is that the EVS plays an essential role in adaptive coding during natural behaviors such as locomotion. In this view, peripheral vestibular sensory encoding is adaptively modulated during locomotion to preserve the signaling of unexpected stimuli by vestibular nerve afferents (reviewed in refs. ^2,10^). Results from prior studies in non-mammalian vertebrate models have provided support for this proposal. For instance, vestibular afferents in toadfish display an increase in mean firing rate and reduction in sensitivity to passive vestibular stimulation when preparing an escape response^11,12^. Likewise, vestibular afferents in semi-isolated in-vitro larval *Xenopus* display an overall reduction in sensitivity to passive vestibular stimulation during bouts of fictive locomotion induced via applied electrical stimulation^13^. Accordingly, these findings have led to proposal that the EVS functions to increase the linear range of afferent responses by increasing the mean firing rate and decreasing sensitivities during locomotion (reviewed in refs. ^2,10,14^).

The location and connectivity of efferent vestibular neurons within the brainstem nuclei are well conserved across species, suggesting a common function in the evolutionary transition from non-mammalian to mammalian vertebrates^10^. Yet, the proposal that the EVS plays a role in adaptive coding during voluntary head movements is at odds with the results of neurophysiological studies in primates showing no difference in vestibular afferent encoding of active orienting head movements and comparable passive head movements^15–17^. Importantly, however, these prior studies did not record afferent responses during active locomotion. Indeed, reports that human subjects experience more stable posture and gaze during running than during walking are cited as evidence to support the view that the EVS transmits locomotor-related information to the vestibular sensory periphery that mediates adaptive coding across vertebrate classes^18–22^. Thus, to date, whether neural encoding by the vestibular periphery is altered during primate locomotion remains an open question.

Accordingly, to directly address this question we recorded from vestibular afferents during self-generated vestibular stimulation experienced during natural voluntary locomotion. Recordings were made from individual semicircular canal and otolith afferents that sense the angular and linear head acceleration, respectively. We found that the responses of individual vestibular afferents in macaque monkeys were unchanged during locomotion regardless of the organ of origin (i.e., semicircular canal or otolith) or the regularity of afferent response. Interestingly, the most sensitive afferents (i.e., irregular otolith afferents) demonstrated an increase in mean firing rate during the running versus walking and passive conditions. However, through a combination of mathematical modeling and nonlinear analysis, we establish that a static nonlinearity in the afferent input-output relationship leads to this effect and that a unified model describes the responses of all classes of afferents across all conditions. Taken together, our results reveal that that head motion information is relayed by individual afferents from the vestibular periphery to higher brain centers in a context-independent manner. Thus, our findings do not provide evidence for the long-standing hypothesis that the EVS plays a role in adaptive coding by modulating vestibular afferent coding in a context-dependent manner during voluntary behaviors such as locomotion. Instead, we speculate that context-dependent modulation occurs centrally, in a pathway-specific manner, to ensure that coding is consistent with behavioral goals during locomotion.

## Results

**Fig. 1:**
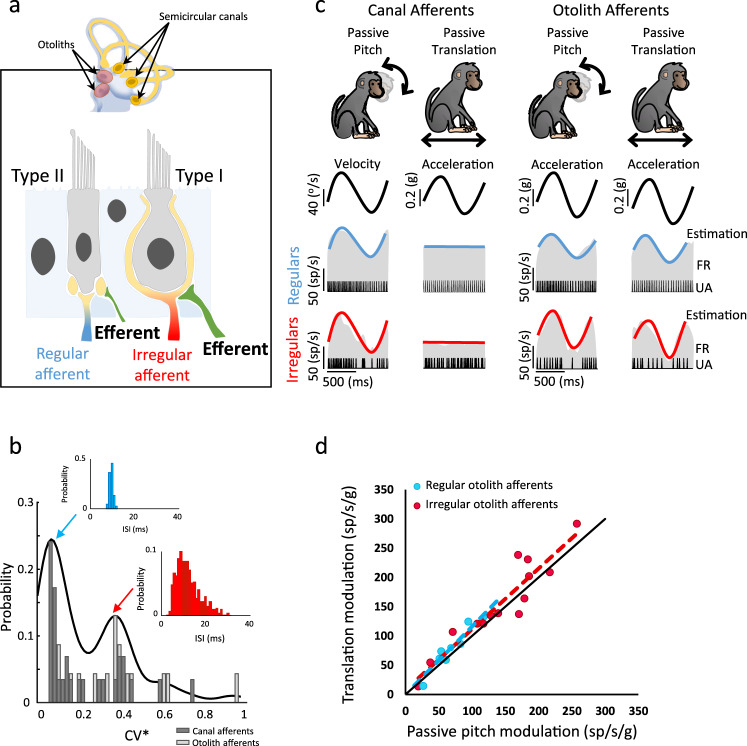
Characterization of vestibular afferents. **a** The vestibular labyrinth comprises five sensory organs: the three semicircular canals and the two otoliths. Within the sensory epithelia of each sensory organ (indicated by arrows) are the receptor cells, which in mammals comprise two types of hair cells: cylindrical type II and flask-shaped type I hair cells. Both canal and otolith afferent fibers are classified on the basis of the regularity of their resting discharge, and in general, irregular afferents (red) preferentially transmit information from the type I hair cells, whereas regular afferents (blue) preferentially transmit information from type II hair cells. The vestibular efferent system (green) consists of a group of neurons located in the brainstem neighboring the abducens nucleus, which projects back out to the vestibular periphery. **b** Bimodal distribution of the normalized CV (CV*) for all recorded afferents (Hartigan’s dip test*, p* = 0.04, *p* = 0.02). Inset shows interspike interval distribution for an example regular (blue) and an example irregular (red) vestibular afferent. **c** Characterization protocol for vestibular afferents. Semicircular canals afferents encode head velocity during head pitch but not during body translation whereas otoliths afferents encode linear head acceleration during both protocols. **d** Otolith afferents have similar sensitivity to passive translation and passive pitch (regression slope: regular otolith afferents: *p* = 2.1 × 10^−4^, CI = 0.42 VAF: translation = 0.92 ± 0.03, passive pitch = 0.95 ± 0.05; Irregular otoliths: *p* = 1.0 × 10^−6^, CI = 0.25, VAF: translation = 0.82 ± 0.04, passive pitch = 0.89 ± 0.06). Source data are provided as a Source Data file.

Individual vestibular afferents (*n* = 55) were first characterized during passive rotations and linear translations. Recorded units comprised 32 vertical semicircular canal and 23 otolith afferents, which were sensitive to rotation alone or the gravito-inertial forces generated by both rotational and translational stimulation, respectively. Regardless of their organ of origin (Fig. 1a), vestibular afferents display a wide range of resting discharge in the absence of movement (CV*, see Methods). Distributions of CV* values were bimodal for both our semicircular canal and otolith afferent populations (Fig. 1b, *p* = 0.04, *p* = 0.02, Hartigan’s dip test). Accordingly, afferents were classified as either irregular or regular, consistent with previous studies (reviewed in refs. ^2,23^). Figure 1c shows the responses of four example afferents. The regular and irregular semicircular canal afferents responded to rotational but not translational head motion. In contrast, the otolith afferents responded equivalently to the net gravito-inertial forces experienced in both conditions (Fig. 1d), as was expected since the gravito-inertial forces produced in each are indistinguishable^24^.

To next establish whether the responses of vestibular afferents were altered during locomotion, we recorded the activities of these same individual afferents during walking and running (see Methods, Supplementary Fig. 1). Prior studies in lower vertebrates, which have been taken as support for the idea that the vestibular efferent pathway modulates peripheral coding during locomotion. (e.g., toadfish^11^, larval *xenopus*^13^), quantified and compared vestibular afferents responses across conditions by computing mean firing rates and peak-to-peak modulation sensitivities. Accordingly, we first analyzed the responses of primate vestibular afferents using this same approach (see Methods). Further, more irregularly discharging afferents are preferentially sensitive to experimentally applied stimulation of the vestibular efferent pathway^6,9^, thus we specifically examined whether locomotion might preferentially alter the responses of irregular afferents.

### Semicircular canal afferents display comparable modulation and baseline activity during passive motion and locomotion

**Fig. 2:**
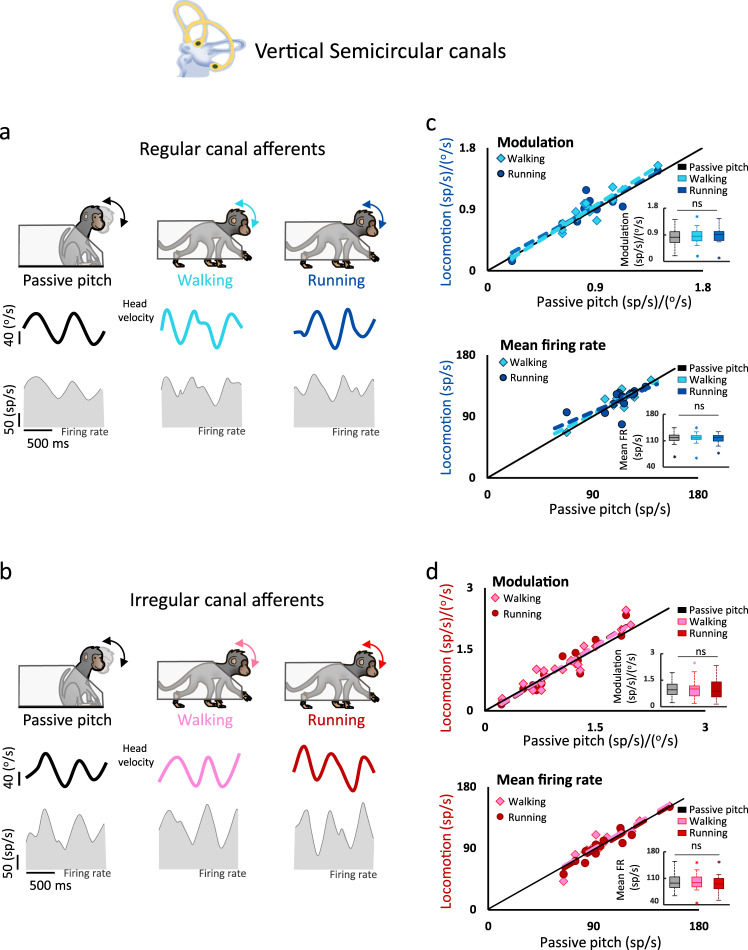
Semicircular canal afferents similarly respond to head velocity during passively applied pitch and locomotion. **a**, **b** Semicircular canal afferents robustly respond in the passive pitch, walking, and running conditions. Bottom panels show the firing modulation (shaded area) of an example regular afferent (**a**) and example irregular afferent (**b**) in each condition. **c** Population comparison of regular semicircular canal afferent responses during locomotion and passive stimulation (*n* = 15). Neuronal modulation (top panel) as well as mean firing rates (bottom panel) were comparable across all conditions, as shown by the slopes of the regressions (top panel*;* walking: *p* = 3.9 × 10^−8^, slope = 1.1, CI = ±0.21 and running: *p* = 7.0 × 10^−6^ slope = 0.97, CI = ±0.28; bottom panel; walking: *p* = 3.0 × 10^−6^, slope = 1.1, CI = ±0.2; running: *p* = 0.02, slope = 1.1, CI = ±0.35; insets*:* ANOVA, *F*_(2,28)_ = 1.2, *p* = 0.31 and *F*_(2,28)_ = 0.38, *p* = 0.96 for modulation and mean firing rate). **d** Population comparison of irregular semicircular canal afferent responses during locomotion and passive stimulation (*n* = 17). Response modulation (top panel) as well as mean firing rates (bottom panel) were similarly comparable across conditions (top panel: walking: *p* = 2.4 × 10^−8^, slope = 1.1, CI = ±0.22 and running: *p* = 3.0 × 10^−8^, slope = 1.1, CI = ±0.21; bottom panel: walking: *p* = 1.3 × 10^−8^, slope = 1.05, CI = ±0.19 and running: *p* = 1.6 × 10^−7^, slope = 1.1, CI = ±0.19; inset*:* ANOVA *F*_(2,32)_ = 0.16, *p* = 0.85 and *F*_(2,32)_ = 1.7, *p* = 0.20 for the modulation and mean firing rate). For all boxplots, the central mark indicates the median, the middle box indicates the 25th and 75th percentiles and the whiskers extend to the most extreme data points not considered outliers. Source data are provided as a Source Data file.

Figure 2 illustrates the responses of semicircular canal afferents. The example regular and irregular afferents shown in Fig. 2a, b were typical in that they responded robustly during walking as well as during comparable passive stimulation. We also investigated whether vestibular afferent responses might be more strongly altered during running than walking, as suggested by previous human studies reporting more stable posture and gaze during the former (e.g., refs. ^19,21^). However, we likewise found that afferents continued to robustly respond to head motion during running (Fig. 2a, b, right panels). Quantification of responses during the walking, running, and passive conditions are summarized for our regular and irregular afferent populations in Fig. 2c and d. Overall, we found no change in the modulation of regular canal afferents during walking and running relative to the passive condition (Fig. 2c; inset: *p* = 0.31). Comparable results were obtained from our analysis of irregular canal afferent response (Fig. 2d; modulation; inset: *p* = 0.85). Finally, we likewise found no change in mean firing rate of either regular (Fig. 2c; inset: *p* = 0.96) or irregular (mean firing rate; inset: *p* = 0.20) canal afferents across conditions. Thus, mean firing rates and modulation sensitivities of semicircular canal afferents were comparable during walking, running, and passive conditions.

### Otolith afferents with higher intrinsic discharge variability show an increase in mean firing rate activity during running, compared to walking and passive conditions

**Fig. 3:**
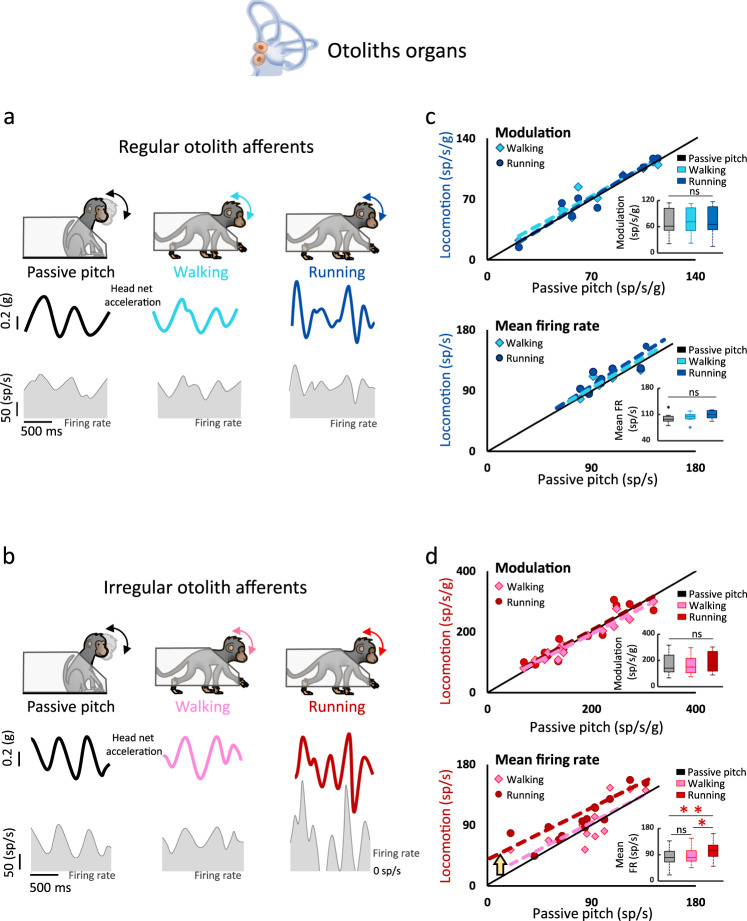
Irregular otolith afferents demonstrate an increase in mean firing rate during running versus walking/passive conditions. **a,** **b** Regular and irregular otolith afferents robustly respond in the passive, walking and running conditions. Bottom panels show the firing modulation (shaded area) of an example regular afferent (**a**) and an example irregular afferent (**b**) in each condition. **c** Population comparison of regular otolith canal afferent responses (*n* = 9). Neuronal modulations (top panel) and mean firing rates (bottom panel) were comparable across all conditions as shown by the slopes of the regressions (top panel*;* walking: *p* = 0.001, slope = 0.95, CI = ±0.27 and running: *p* =2.6 × 10^−4^, slope = 0.93, CI = ±0.35; bottom panel; walking: *p* = 0.03, slope = 1.01, CI = ±0.37; running: *p* = 0.05, slope = 1.07, CI = ±0.28; inset: ANOVA, *F*_(2,16)_ = 0.45, *p* = 0.65 and *F*_(2,16)_ = 2.7, *p* = 0.09 for the modulation and mean firing rate, respectively). **d** Population comparison of irregular otolith afferent responses (*n* = 14). Neuronal modulation was comparable during locomotion and passive stimulation (walking: *p* = 1.3 × 10^−8^, slope = 0.97, CI = ±0.13 and running: *p* = 4.0 × 10^−6^, slope = 0.92, CI = ±0.26, respectively; inset: ANOVA, *F*_(2,26)_ = 1.03, *p* = 0.37). In contrast, mean firing rate was higher (gold arrow) during running compared to passive pitch and walking (walking: slope = 0.84, *p* = 0.001, CI = ±0.30; intercept = 14.6 sp/s, *p* = 0.49; running: slope = 0.77, *p* = 0.001, CI = ±0.30; intercept = 33.7 sp/s, *p* = 0.007; inset: ANOVA, *F*_(2,26)_ = 6.0, *p* = 0.007). For all boxplots, the central mark indicates the median, the middle box indicates the 25th and 75th percentiles and the whiskers extend to the most extreme data points not considered outliers. Source data are provided as a Source Data file.

Individual vestibular efferents innervate multiple end organs, including both the semicircular canals and otoliths (reviewed in ref. ^2^). Nevertheless, there is evidence that overall, the vestibular efferent system more strongly targets the otolith organs^25–27^. This difference, in turn, could lead to preferential modulation of otolith-dependent sensory coding at the level of the vestibular periphery during locomotion. Accordingly, we next compared otolith afferent responses during locomotion and passive head motion (Fig. 3a, b) following the same approach described above for semicircular canal afferents. The example regular afferent (Fig. 3a) was typical in that it continued to respond robustly during walking and running. While this was also the case for the example irregular afferent (Fig. 3b), this afferent displayed a marked increase in mean firing rate during running condition relative to the walking and passive conditions (compare right panels of Fig. 3a, b).

Quantification of responses during the walking, running, and passive conditions are summarized in Fig. 3c, d for our regular and irregular otolith afferent populations. Overall, we found no change in the modulation sensitivities of regular otolith afferents during walking and running relative to the passive condition (Fig. 3c top: inset: *p* = 0.65). Likewise, their mean firing rates were unchanged in both locomotion conditions (Fig. 3c bottom: inset: *p* = 0.09). Similarly, both response measures were unchanged for irregular otolith afferents in the walking versus passive head motion conditions, and the response modulation of irregular otolith afferents was comparable across conditions (Fig. 3d top panel: inset: *p* = 0.37). In contrast, however, the mean firing rate of this afferent displayed a significantly increase during running, as can be seen in Fig. 3d (bottom panel) where most points lie above the identity line (inset: *p* = 0.007). Thus, mean firing rates and modulation sensitivities of all semicircular canal afferents and regular otolith afferents were comparable during walking, running, and passive conditions. However, irregular otolith afferents displayed a significant increase in mean firing rate during running relative to the walking and passive conditions (Fig. 3d).

### Taking response nonlinearity into account explains the observed increase in irregular otolith afferent mean firing rate during running

**Fig. 4:**
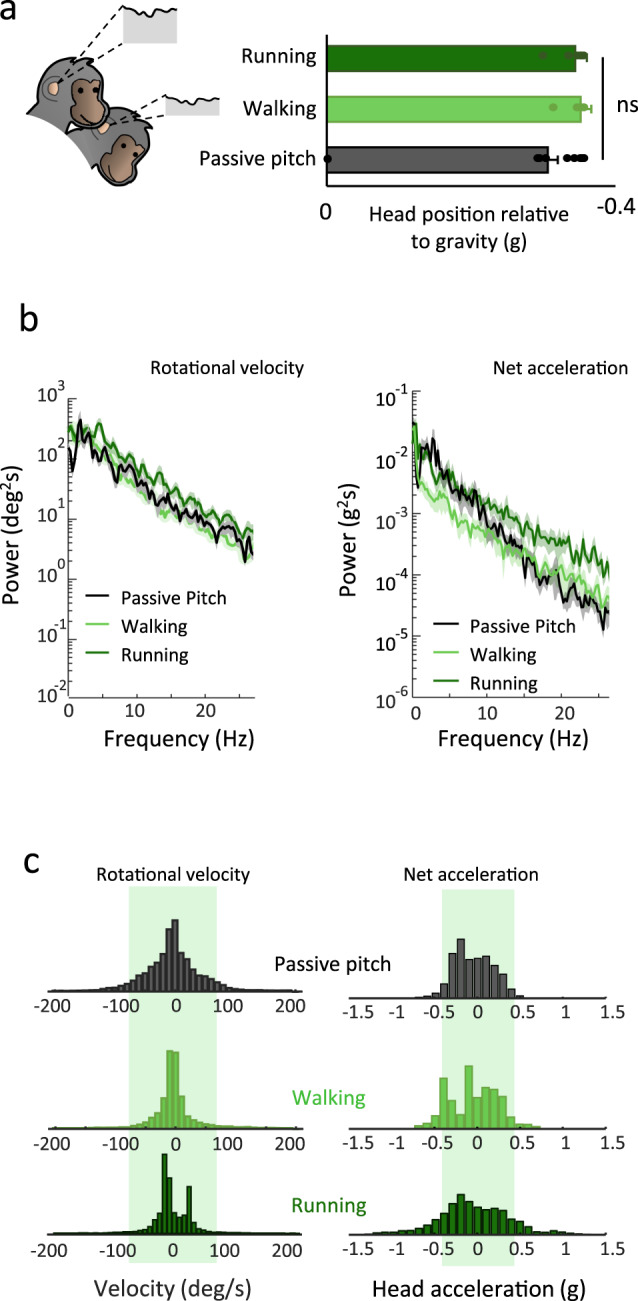
Comparison of head motion during locomotion and passive conditions. **a** Average head position was similar in the three conditions (mean ± 1 STD, ANOVA, *F*_(2,26)_ = 1.9, *p* = 0.17). **b** The spectral power of rotational pitch velocity was comparable during passively applied stimulation and walking, but higher during running (left panel). Similarly, the mean spectral power of net acceleration was greater during running than during passively applied pitch and walking (right panel) which did not differ from each other. Shaded area represent ± 1 STD. **c** Comparison of probability distributions of motion amplitude across conditions. The shaded green areas represent ± 1.5 STD of movement amplitudes generated by passive stimulation. For the passive pitch, walking, and running conditions, 3%, 2 and 4% of the rotational velocity values were outside of this range, respectively. In contrast, the probability of linear acceleration reaching values beyond ±1.5 SD of the walking-matched passive condition (shaded green area) was more than two times greater during running compared to the walking and passive conditions (15% versus 5 and 3%, respectively)—corresponding to significantly higher kurtosis and standard deviation values in this condition (all *p*-values < 0.03). Source data are provided as a Source Data file.

The above finding that irregular otolith afferents demonstrated an increase in mean firing rate during running could be interpreted as support for the long-standing hypothesis that the EVS functions to mediate adaptive sensory coding in the vestibular periphery during locomotion^2^. However, there are two alternative explanations for these results. First, because the otoliths are sensitive to net acceleration (i.e., gravity as well as linear acceleration), their firing rates will be modulated as a function of changes in head position relative to the gravity vector (Fig. 4a). Thus, one possibility is that on average, the head was oriented differently relative to gravity in running versus walking and pitch conditions, which in turn would result in a change in the mean firing rate for the irregular otoliths. To test this possibility, we compared the mean head position relative to the gravity across conditions. However, we found no difference in the average orientation of the head relative to gravity in the running versus walking and naturalistic pitch conditions (ANOVA, *p* = 0.17).

A second possibility is that the running condition preferentially evoked response nonlinearities (e.g., saturation and inhibitory cutoff) in the responses of irregular otolith afferents. Notably, these afferents are exceptionally sensitive to head motion, demonstrating significantly higher modulation gains than their regular counterparts^28,29^. The applied passive-pitch stimuli were designed to have head movement trajectories comparable to those experienced during active walking (see *Methods*), so running should result in more vigorous vestibular stimulation. To test this possibility, we compared the vestibular stimulation experienced in each condition. We found that indeed the spectral power of both head net acceleration and rotational pitch velocity was higher during running (Fig. 4b). Accordingly, plotting the cumulative histogram for each of these spectra resulted in a significantly smaller area under the running curve relative to the two other conditions (Supplementary Fig. 2; *p* = 5.9 × 10^−5^ and *p* = 9.2 × 10^−7^, for rotational velocity and net acceleration, respectively). In addition, the amplitude of the net acceleration experienced during running reach greater values than during passive-pitch and walking condition (Fig. 4c). On average, 15% of the net acceleration values were outside of 1.5 standard deviation, which was significantly greater than that observed of the two other conditions (i.e., 5 and 3%, respectively, Supplementary Fig. 2b, *p* = 8.5 × 10^−5^). This was not observed for the rotational velocity (*p* = 0.13).

**Fig. 5:**
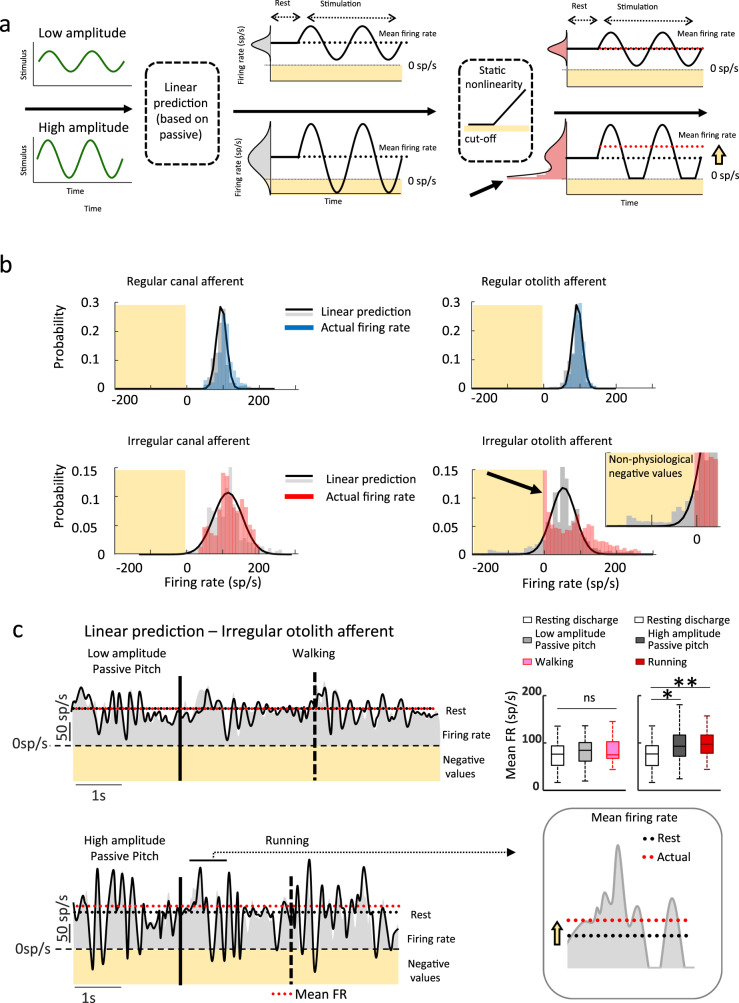
Irregular otolith afferents show nonlinear responses during running. **a** A hypothetical linear neuron’s response to low (top) and high (bottom) amplitude stimulation. In response to high amplitude stimulation, the model predicts negative values resulting in physiological inhibitory cutoff that causes an increase in overall mean firing rate relative to the afferent’s resting discharge. The higher probability of having negative values that result in inhibitory cutoff increases the mean firing rate. **b** Actual and linear model-predicted probability distributions all afferent classes. The passive-based linear model (Eq. 3) predicts impossible negative firing rates (yellow shaded area; in panels **a**, **b**, and **c**). The probability of zero firing rate (black arrow) was significantly higher for irregular otolith afferents than the other classes of afferents (one-way ANOVA, *F*_(3,45)_ = 13.45, *p* = 2.5 × 10^−6^). **c** Actual firing rates and linear model predictions across conditions. As shown in panel **b**, neuron’s nonlinear response (i.e., inhibitory cutoff) resulted in an increase in mean firing rate during high amplitude passive stimulation and running (VAF = 0. 35, and 0.37) for the high amplitude passive pitch and running respectively, while this was not the case for the lower-amplitude stimulation (i.e., walking and walking-matched passive pitch, (VAF = 0.89 and 0.86, respectively). Top right insets: Population-averaged mean firing rates were significantly higher than resting discharges during high amplitude passive motion and running (ANOVA, *F*_(4,52)_ = 7.8, *p* = 0.002), which did not differ from each other (*p* = 0.85). Mean firing rate and resting discharge were comparable for low-amplitude passive pitch and walking were comparable (*p* = 0.20). For all boxplots, the central mark indicates the median, the middle box indicates the 25th and 75th percentiles and the whiskers extend to the most extreme data points not considered outliers. Source data are provided as a Source Data file.

Thus, the net head accelerations experienced during running did, in fact, result in more vigorous otolith stimulation compared to the passive and walking conditions. Figure 5a illustrates the encoding of a low (*top*) and more vigorous high (*bottom*) amplitude linear acceleration stimulus by a hypothetical highly sensitive neuron. The neuron’s response remains in the linear range for low but not high amplitude stimulation where it is driven into inhibitory cutoff (static nonlinearity, Fig. 5a). Notably, since it is not physiologically possible for neurons to fire at negative rates, the hypothetical neuron’s firing rate extends into a region of the input-output relationship that elicits nonlinearity and generates a spike in its firing rate probability distribution at zero sp/s (black arrow). Additionally, because of this nonlinearity, the mean response during stimulation will be higher than its resting discharge (yellow arrow). Thus, if irregular otoliths afferents were preferentially driven into a nonlinear stimulus-encoding regime in response to the more robust stimulation experienced during running, we predict that (1) the smaller range of afferent firing rates evoked during walking are contained in a region for which the central vestibular neuron input-output relationship is approximately linear, (2) the greater range of afferent firing rates evoked during running extend into the nonlinear region of the input-output relationship, and as a result, (3) irregular otoliths afferents responses will specifically show a higher probability of inhibitory cutoff, resulting in a spike in the likelihood of a zero sp/s firing rate during running.

To test our hypothesis, we first compared the probability distributions of afferent firing rates during walking and running (Fig. 5b). Our example irregular otolith afferent displayed a spike in its probability of firing at zero sp/s (Fig. 5b, denoted by the arrow, bottom right plot) that was not evident in the other 3 example afferent response distributions. Quantification across our population of afferents revealed that an irregular otolith afferent’s firing rate was significantly more likely to be zero during running compared to the 3 other afferent classes (*p* = 2.5 × 10^−6^).

In contrast, the probability of zero sp/s firing during walking was low and comparable across all four afferent classes (Supplementary Fig. 3a; *p* = 0.56). Accordingly, these findings confirmed our predictions, and establish that irregular otoliths afferents specifically show a spike in the probability of firing at zero sp/s during running. Thus, highly sensitive irregular otolith afferents are indeed characterized by a strong nonlinearity (inhibitory cutoff) that is primarily elicited by the more vigorous stimulation experienced during running.

It then follows that the modulation of irregular otolith afferents cannot be accurately represented by linear models during running. Importantly, however, the assumption of linearity is inherent to prior interpretations of analyses only comparing mean firing rates and peak to peak modulation across conditions (e.g., refs. ^11,13^). Figure 5c shows the responses of our example irregular afferent during walking and running (gray shaded traces). As expected, we found that this model (black trace) frequently predicted negative firing rates (shaded gold area), which are of course outside the range of physiological possible firing rates during running but not walking. During these intervals, the afferent instead displayed inhibitory cutoff and thus demonstrated an increased probability of firing at zero sp/s. Similar results were observed for passive stimulation with comparable dynamics to walking and running conditions, termed low and high amplitude passive motion, respectively (compare top and bottom panels). The observed increased probability of firing at zero sp/s resulted in higher average firing rates for irregular otolith afferents for the more vigorous stimulation experienced during running and comparable passive stimulation, compared to the walking and comparable passive stimulation conditions (compare superimposed dashed red and dashed black lines, Fig. 5c). This is because the cutoff nonlinearity at zero sp/s effectively shifts the population distribution to more positive values.

Indeed, these results were consistent in our population of irregular otolith afferents population (Fig. 5c: bar plots). Specifically, mean firing rates were significantly higher than resting discharges during high amplitude passive motion and running (*p* = 0.002) which did not differ from each other (*p* = 0.85). In contrast, the mean firing rate and resting discharge were comparable for low-amplitude passive pitch and walking (*p* = 0.20). For completeness, we computed this same analysis on our other three groups of afferents and found no difference between mean firing rates and resting discharges and across all testing conditions (Supplementary Fig. 3b, c; all *p-*values > 0.07). As such, our results indicate that the observed increase in the mean firing rate of irregular otolith afferents during running was due to nonlinear responses evoked by the higher amplitude vestibular stimulation.

### Building a unified model of vestibular afferent responses to locomotion and passive stimulation

**Fig. 6:**
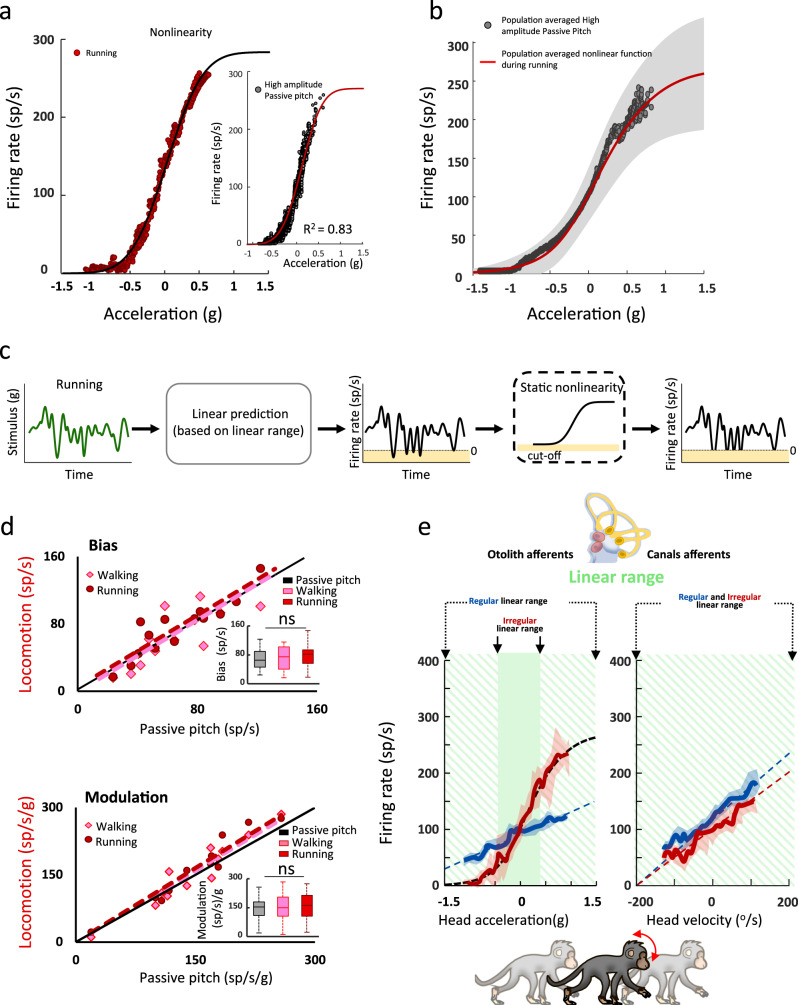
Taking into account response nonlinearity reveals that irregular otolith afferents similarly encode locomotion and passive motion. **a** During running, the example irregular otolith afferent’s response as a function of acceleration was well fit by a sigmoid (black sigmoid). The same nonlinearity accurately described this afferent’s responses during high amplitude passive head motion (inset, R^2^ = 0.83). **b** The population-averaged response as a function of acceleration was comparable during high amplitude passive head motion (gray dots) and running (red sigmoid). Shaded area represents ± 1 STD of the population average nonlinear function during running. **c** Schematic of the linear-nonlinear cascade model. In this model the output firing rate is calculated by first linearly filtering the input stimulus and then passing the resulting linear prediction through a static nonlinear function. **d** Top panel*:* The bias estimated using the linear-nonlinear cascade model was comparable between conditions (walking: slope = 1.01, *p* = 0.002, CI = 0.45, intercept = 5.9 sp/s, *p* = 0.6; running: slope = 1.06, *p* = 3.2 × 10^−4^, CI = 0.35, intercept = 6.9sp/s, *p* = 0.07). Inset: population-averaged bias values for each condition (ANOVA, *F*_(2,26)_ = 1.7, *p* = 0.25). Bottom panel*:* The modulation of irregular otolith afferents is comparable across conditions when using a nonlinear model that accounts for the afferent’s responses to large-amplitude head accelerations (walking: slope = 1.06, *p* = 3.0 × 10^−6^, CI = 0.25; running: slope = 1.09, *p* = 2.8 × 10^−7^, CI = 0.20; *inset:* ANOVA, *F*_(2,26)_ = 2.1, *p* = 0.14). **e** Schematic showing that the responses of semicircular canal afferent and regular otolith afferent responses remain in their linear coding range during all conditions (striped green area), but irregular otolith afferent responses extend into a nonlinear coding range during running. Shaded areas represent ± 1 STD of the population average firing rate. For all boxplots, the central mark indicates the median, the middle box indicates the 25th and 75th percentiles and the whiskers extend to the most extreme data points not considered outliers. Source data are provided as a Source Data file.

To characterize the static nonlinearity in the irregular otolith afferent responses identified above, we next plotted the firing rate as a function of the time-shifted head acceleration for each afferent during running (Fig. 6a) and high amplitude passive motion (Fig. 6a, inset) and found the relationship was well fit by a sigmoidal function. Importantly, the same sigmoidal function describing the irregular otolith afferents responses during running well described their response in the high amplitude passive condition (Fig. 6b). Accordingly, we next explicitly tested if, by properly accounting for this nonlinearity, we could use a unifying model to describe afferent responses across all passive and active conditions. To do this, we used a linear-nonlinear (LN) model (Fig. 6c and Supplementary Fig. 4a^30,31^). Specifically, the linear stage (Eq. 3) of the LN model was estimated over the linear range of each irregular otolith afferent’s response during running (Supplementary Fig. 4b and inset, green shaded areas). The nonlinear stage of the LN model was then estimated by plotting the actual firing rate response as a function of the linear prediction and by fitting a sigmoidal function (Supplementary Fig. 4a). Note as expected, the linear model predicted negative values corresponding to actual firing rates of 0 sp/s (i.e., cutoff).

Overall, this LN model predicted the responses of irregular otolith afferents much more accurately (>2-fold increase in VAF) compared to linear models which were (i) estimated based on a given afferents response to walking-matched amplitude passive stimulation, (ii) optimized to increase the resting bias relative to the passive condition, and/or (iii) optimized to increase the resting bias and decrease modulation (Supplementary Fig. 5) for both the running and high amplitude passive conditions (*p* = 3.23 × 10^−32^ and *p* = 1.15 × 10^−10^, respectively). However, this was not the case for the three other classes of afferents, for which we observed that the nonlinear model did not provide a better fit than the linear model (Supplementary Fig. 6; (*p* = 0.41, *p* = 0.58, *p* = 0.28 for the regular and irregular canal and the regular otolith afferents, respectively).

Importantly, our LN based analysis further revealed that the bias, as well as modulation sensitivity estimated for running, was actually comparable to that observed for walking and walking-matched passive condition. Figure 6d summarizes these observations for our irregular otolith afferent population. Notably, this contrast with our initial findings regarding the mean firing rate (Fig. 3d, bottom). Thus, taken together, our results reveal that once the static nonlinearity in the afferent input-output relationship is properly accounted for, a unified model can describe the responses of all classes of afferents across running, walking, and passive stimulation conditions. Specifically, as summarized in Fig. 6e, semicircular canal afferents and regular otoliths afferents remain in their linear coding range across the tested conditions (Supplementary Fig. 7), while the responses of irregular otolith afferents extend into their nonlinear coding range during running (Fig. 6a).

## Discussion

Our central finding is that head motion information is relayed faithfully from the vestibular periphery to higher brain centers during primate locomotion. Through a combination of mathematical modeling and nonlinear analysis, we establish that vestibular afferent responses can be explained using a unified model irrespective of context. This conclusion is based on recordings obtained from the same single afferents in *rhesus* monkeys across active and passive conditions including walking, running and comparable passive stimulations. Specifically, we demonstrate that both individual semicircular canal and otolith afferents in the vestibular periphery encode a robust and unaltered representation of head motion during locomotion. Notably, this result contrasts sharply with the conclusions of prior studies in non-mammalian models of the vestibular^12,13^ and evolutionarily related lateral line^32–34^ systems. As discussed below, we speculate that a benefit of such context-independent coding at the level of the mammalian vestibular periphery is the ability to provide unambiguous input to target neurons in central vestibular pathways. It is then at this next stage of processing that pathway-specific modulation underlies distinctive behaviorally dependent gating of the vestibulo-ocular reflex (VOR) versus vestibulospinal reflexes and ascending thalamocortical pathways.

The mammalian vestibular efferent system sends bilateral projections to the vestibular periphery and does not make synaptic contacts with neurons within the vestibular nuclei (reviewed in ref. ^35^). As a result, it can only modulate the afferent input to the vestibular nucleus by inducing significant changes in afferent responses (e.g., in contrast to the presynaptic control observed in dorsal root ganglion). Accordingly, studies have been recorded from individual vestibular afferents to gain insight into the role of the vestibular efferent system. Experimentally applied stimulation of the vestibular efferent system preferentially modulates more irregularly discharging semicircular canals and otoliths afferents in mammals^6,9^. Irregular afferents have higher sensitivities than regular afferents and are thus more prone to nonlinear responses (e.g., inhibitory cutoff and excitatory saturation) during dynamic head motion^17,31^. Thus, it has been proposed that it would be theoretically beneficial for the vestibular efferent pathway to reduce irregular afferent modulation during locomotion to preserve the signaling of unexpected stimuli (reviewed in refs. ^2,10^). Additionally, human subjects with and without peripheral vestibular loss exhibit more stable posture and gaze during running than during walking. This observation has led to the further proposal that EVS additionally transmits motor-related signals to the periphery that improve motor performance during locomtion^5,18–22^.

Our present findings, however, do not provide support for either idea. Instead, we found that irregular, as well as regular vestibular afferents, faithfully encode a robust and unaltered representation of head motion, during running as well as walking. Interestingly, prior studies have likewise shown no change in vestibular afferent responses during active head movements produced by activation of the neck musculature^11–13^. Indeed as discussed below, such a context-independent peripheral coding strategy is a necessary condition for the context-dependent vestibular coding that occurs in the parallel pathways that mediate the vestibulo-ocular and vestibulospinal reflexes; a central-based pathway-specific cancellation strategy is required to ensure that the efficacy of the VOR remains intact during locomotion, while simultaneously suppressing the stabilizing mechanisms of the vestibulospinal reflex when the goal is to actively locomote through space^36,37^.

The VOR is vital for effectively stabilizing our visual axis of gaze relative to space during everyday activities (reviewed in ref. ^1^). In particular, this vestibular-driven reflex is required for gaze stability during active behaviors such as locomotion because the dynamics of VOR eye movements make effective compensation possible over the frequency range of natural head movements^38,39^. In contrast, the dynamics of the visually driven pursuit and optokinetic systems are too slow to compensate for such dynamic head motion (see ref. ^24^). Accordingly, during locomotion, individuals with bilateral vestibular hypofunction demonstrate significant gaze instability relative to healthy controls^40^. Thus, in the context of the VOR’s functional goal of stabilizing gaze, it is logical and indeed necessary that the vestibular afferents transmit a robust and accurate representation of head motion to central VOR pathways—regardless of whether head motion is passively applied or the result of locomotion. In fact, this robust and accurate representation of head motion underlies the VOR’s ability to provide a stable gaze as we walk or run. Therefore, we predict that the vestibular sensitivities of central VOR neurons, like their vestibular afferent input, will also be robust and unchanged to maintain stable gaze during locomotion.

At the same time, vestibulospinal reflex pathways are essential for the maintenance of posture and balance (reviewed in ref. ^1^). However, in contrast to the VOR, the vestibulospinal reflex is counterproductive when the behavioral goal is to actively move through space – as during locomotion and other voluntary behaviors (reviewed in ref. ^1^). Nonetheless, the present study shows that peripheral vestibular coding is unaltered during voluntary locomotion. This is also the case for voluntary orienting head movements generated by the neck musculature^15,17^. Thus, vestibular afferents transmit a context-independent representation of head motion to the central vestibulospinal reflex pathways. We speculate that vestibular signals that are the consequence of voluntary locomotion are instead cancelled at this first stage of central processing in vestibulospinal pathways during locomotion, as has been shown to be the case for voluntary orienting head movements^41–43^. Further, it has been established that these same central neurons selectively respond to unexpected head motion during active orienting head movements^41,42^. Thus, the observed cancellation is not due to a general gain change in vestibular peripheral transmission. We speculate that unexpected head motion will likewise be encoded during locomotion to ensure corrective changes in postural tone to such perturbations. Indeed, while single-unit experiments are ultimately required to directly test this prediction, recent studies provide evidence for the suppression of vestibular balance stabilizing mechanisms during human locomotion^44,45^.

It is noteworthy that no prior study, in any species, had directly tested peripheral motion sensing by the vestibular system during active locomotion. Here we recorded the activity of individual vestibular afferent fibers in *rhesus* monkeys as they actively locomoted through space. In contrast, influential studies in lower vertebrates that are widely considered to support this idea (i.e., toadfish^11^, larval *xenopus*^13^), did not actually record the afferent activity during voluntary head movement generated by active locomotion^5^. Instead, these studies used much lower-amplitude passive vestibular stimulation (<60 degs/s) compared to active head movements generated by monkeys during locomotion in our present study (150 degs/s). Overall, our present results provide direct evidence against the idea that the vestibular efferent system modulates peripheral motion sensing during active locomotion in primates. It is nevertheless possible the EVS could function to modulate peripheral vestibular feedback during more dynamic movements such as that experienced playing soccer (humans^38^) or jumping from cage to cage (monkeys^39^). In a prior study, we demonstrated that sustained steps of high constant-velocity rotation (>300 deg/s) can also evoke efferent-mediated responses in vestibular afferents^9^. However, such sustained vestibular stimuli are unnatural, and further, the observed efferent activation was characterized by much slower dynamics than natural vestibular stimuli^38,39^. Thus, future studies recording from afferents in freely moving animals during such challenging dynamic behaviors will be required to test this possibility.

One possible explanation for the differences observed here and in prior studies of non-mammalian vertebrates is that neural strategies changed over the course of evolution to produce species-specific behaviours. In non-mammalian vertebrates such as *Xenopus*, CPGs within the spinal cord generate rhythmic locomotor movements during swimming^46^. Locomotor CPGs have also been identified in the spinal cords of numerous mammalian vertebrate species including mice, rats, rabbits, and cats. Yet, to date, the question of whether and where CPGs are located in the primate spinal cord remains open (reviewed in ref. ^47^). Stimulation experiments in monkeys and humans with spinal cord transection have been further unable to identify a central pattern generator for locomotion (*macaque*:^48^, *marmoset*:^49^, *human*^50^). Instead, the flexible, adaptive locomotor patterns of primates appear to be predominantly driven by descending supraspinal inputs including cortical commands. Consistent with this idea, recent single-unit recording experiments have demonstrated that motor cortex neurons robustly fire during locomotion in a highly context-dependent manner^51^. Thus, a fundamental difference in the evolution of the locomotion circuitry could then play a role in explaining why reafferent vestibular signals are not altered at the level of the sensory periphery during primate locomotion.

Another likely possibility, which is not mutually exclusive with the evolutionary explanation above, is that the vestibular efferent system plays a significant role in modulating peripheral vestibular sensing over a longer time course—for example during development and aging. The experiments of Chagnaud and colleagues^13^ focused on larval rather than mature *Xenopus*. To date, it remains unknown whether transmitted spinal corollary discharge signals continue to alter vestibular peripheral coding in mature *Xenopus* that have developed their adult locomotor strategy^52^. We speculate that during development the mammalian vestibular efferent system serves an important role in balancing the input from the two labyrinths and calibrating central pathways—analogous to that described for the auditory efferent system^53^. In mature primates, the population responses of regular and irregular vestibular afferents remain unchanged even following the complete removal of peripheral vestibular input from the other side (i.e., unilateral labyrinthectomy). Yet, a small but significant shift has been reported in the overall distribution of afferents towards greater discharge irregularity^17^. Furthermore, mutant mice that develop from the beginning with a targeted deletion of the neuroactive peptide αCGRP gene display a marked reduction in vestibular function as adults^54^. Since αCGRP colocalizes with key components of the vestibular efferent system^3,55^, the observed impairment is consistent with the EVS playing a role in the normal development of vestibular pathways. It is also noteworthy that lesions of the mammalian auditory efferent pathway can both accelerate age-related hearing loss^56^ and reduce protection against noise exposure (reviewed in ref. ^57^). Accordingly, we further speculate that lesions to the mammalian vestibular efferent pathway in mammals would serve a similar neuroprotective role regarding age-related peripheral vestibular impairment^58,59^ and noise-induced damage (reviewed in ref. ^60^). Future experiments, however, will be required to address each of these possibilities.

## Methods

### Lead contact and materials availability

Further information and requests for resources should be directed to and will be fulfilled by the Lead Contact, Kathleen Cullen (kathleen.cullen@jhu.edu). This study did not generate new unique reagents.

### Experimental model and subject details

Animal experimentation: All experimental protocols were approved by the McGill University Animal Care Committee (#2001–4096) and were in compliance with the guidelines of the Canadian Council on Animal Care. The vestibular afferent recording experiment was conducted in two male cynomologus monkeys (*Macaca Fascicularis*, 6 years old, 4 and 5 kg, respectively). The animals were housed in pairs on a 12 h light/dark cycle. They were brought to the laboratory, about three times a week, for ~2 h recording sessions. All animals had participated in previous experiments in our laboratory but all of them were in good health condition and did not require any medication^61^.

### Method details

#### Surgical procedures

The two animals were prepared for chronic extracellular recording using aseptic surgical techniques described previously^28,61^. Briefly, animals were pre-anaesthetized with ketamine hydrochloride (15 mg/kg im) and injected with buprenorphine (0.01 mg/kg im) and diazepam (1 mg/kg im) to provide analgesia and muscle relaxation, respectively. Loading doses of dexamethasone (1 mg/kg im) and cefazolin (50 mg/kg iv) were administered to minimize swelling and prevent infection, respectively. Anticholinergic glycopyrrolate (0.005 mg/kg im) was also preoperatively injected to stabilize heart rate and to reduce salivation, and again every 2.5–3 h during surgery. During surgery, anesthesia was maintained using isoflurane gas (0.8–1.5%), combined with a minimum 3 l/min (dose adjusted to effect) of 100% oxygen. Heart rate, blood pressure, respiration, and body temperature were monitored throughout the procedure. During the surgical procedure, a stainless-steel post for head immobilization and two stainless-steel recording chambers allowing access to the vestibular nerve were fastened to each animal’s skull with stainless-steel screws and dental acrylic. Craniotomy was performed within the recording chamber to allow electrode access to the brainstem. An 18 mm-diameter eye coil (three loops of Teflon-coated stainless-steel wire) was implanted in one eye behind the conjunctiva.

Following surgery, we continued dexamethasone (0.5 mg/kg im; for 4 days), anafen (2 mg/kg day one, 1 mg/kg on subsequent days), and buprenorphine (0.01 mg/kg im; every 12 h for 2–5 days, depending on the animal’s pain level). In addition, cefazolin (25 mg/kg) was injected twice daily for 10 days. Animals recovered for a minimum of 2 weeks before any experimenting began.

#### Data acquisition

During experiments, monkeys initially sat in a primate chair fixed to a gimbal frame that was mounted onto a linear sled that provided the ability to apply translations along the naso-occipital or inter-aural axes in the horizontal plane along a 3.5 m linear track. The monkey’s head was affixed to a head holder that provided the ability to apply passive head-on-body tilt rotations around the pitch axis prior to locomotion. Once the response of a given afferent had been characterized during the passively applied pitch and translation, the same afferent’s response was recorded while the monkey made active head-on-body tilt rotation around the pitch axis. The floor of the chairwas then removed allowing the monkeys to voluntarily walk and run the length of the linear track quadrupedally, which they had been trained to do. In this condition, the monkey’s head could freely move in sagittal plane (i.e., vertical/naso-occipital axes) and also rotate around the pitch axes. Extracellular single-unit activity was recorded with tungsten microelectrodes (Frederick-Haer Co., Bowdoinham, ME). Linear head and body acceleration were measured using 3-D linear accelerometers (ADXL330Z, Analog Devices, Norwood, MA). Angular head and body velocity were measured using three-axis gyroscopes (QGYR330HA, Analog Devices, Norwood, MA). The linear accelerometers and gyroscopes were firmly attached to the animal’s head post and chair frame. Eye movement, rotational head velocity, and translational head acceleration signals were low-pass filtered at 250 Hz (8-pole Bessel filter) and sampled at 1 kHz.

#### Passive head motion paradigms

Because the afferents in the vestibular nerve were approached through the floccular complex—identified by its eye movement-related activity^15^ monkeys were trained to visually track a target light. Vestibular afferents did not respond to eye movement and showed strong modulation to head movement stimulation. We then quantified the responses of single afferents in the vestibular nerve. We first recorded each afferent’s response during translations generated by passively translating the head-restrained monkey along the naso-occipital and inter-aural axes (1 Hz, ±0.2 g). We then carefully released the brake of the head holder and passively applied head-on-body pitch rotations (1 Hz, ±0.2 g or 40 deg/sec) to measure each afferent’s response to changes in the head orientation relative to gravity and rotational velocity. All afferents described in this study were activated either by pitch movements and/or by translational head movements along at least one of the 2 axes tested. We then tested each afferent’s response to naturalistic passive-pitch head movements whose statistics were comparable to the statistics of head movements during walking (Fig. 4b and c). We found that 32 units were sensitive to pitch rotation alone, while 23 were sensitive to net acceleration during both pitch rotations and translational stimulation. Our semicircular canal afferent dataset comprised *N* = 15 regular and *N* = 17 irregular afferents, and our otolith afferent dataset comprised *N* = 9 regular and *N* = 14 irregular afferents.

#### Active head motion paradigms

Once we had characterized the responses of vestibular afferents during the passive paradigms described above, each afferent’s response was recorded during active head-on-body pitch movements with comparable stimulus statistics to the passive stimulation above while the monkey’s head was released. As previously described^61^ monkeys made these active head-on-body tilt movements for a food reward. Relatively little training was required since monkeys readily generated active tilts within the first day of introducing this task. Our vestibular afferents population was typical in that, as shown by our previous work^16,61^, all afferent classes displayed a similar response to passively applied and actively generated sinusoidal head motion (*p* = 0.29). After a given afferent’s response had been tested during an active pitch, we carefully removed the bottom of the chair so that the monkey could locomote along the linear track. Once the bottom of the chair was removed, monkeys readily and promptly started walking (Supplementary Fig. 1, histogram) along the track. The ends of the track were each mounted on a pivot that when released allowed the experimenter to rotate the monkey by 180 degs so that it could perform repeated trips along the track. For each recorded neuron, the monkey performed two round trips along the track which were pooled together to compute neural response. Additionally, we were able to maintain isolation of the majority of afferents following the walking condition, and then next recorded afferent responses while the monkey ran along the track. Monkeys had been trained to run in response to an auditory cue for a food reward. As illustrated in Supplementary Fig. 1 (inset), the onset and offset of locomotion were defined as the last peak jerk preceding the peak acceleration or the peak deceleration of the body, respectively. The elapsed time between locomotion onset and offset was used to calculate the monkey’s mean velocity. The distribution of mean velocity was bimodal (Hartigan dip test, *p* = 0.01). Consistent with visual reports, the mean velocities of locomotion segments classified as walking fell within the first distribution while those classified as running fell within the second distribution. Furthermore, quantification of the two distributions revealed that mean velocity and peak acceleration was significantly higher for running versus walking (Supplementary Fig. 1, right most panel; t_(54)_ = 13.3, *p* = 1.5 × 10^−8^, t_(54)_, = −11.5, *p* = 3.7 × 10^−9^, respectively). Note that the above locomotion experiments were performed (i) in a well-lit environment and that (ii) the monkey actually travelled (locomoted) across the room—thus the multisensory context was natural in that the animal actually moved through space and experienced complementary vestibular, proprioceptive and visual sensory inputs.

### Analysis of neuronal discharges

Data were imported into the MATLAB (The MathWorks, Natick MA) programming environment for analysis. Estimates of the time-dependent firing rate were obtained by low-pass filtering the spike train using a Kaiser window as previously described^28,62^. The regularity of resting discharge (i.e., in the absence of stimulation) of each afferent was determined by means of a normalized coefficient of variation (CV*) of the interspike intervals (ISIs) recorded during spontaneous activity using the standard method described by Goldberg et al.^63^. Afferents with low values of CV* were classified as regular, whereas those high values of CV* were classified as irregular as in previous studies (cutoff value = 0.15)^16^.

#### Linear models of vestibular afferents responses

A least-squares regression analysis was used to describe the response of each canal afferent to rotational velocity to pitch rotations. As in our previous publication^17^, analysis was restricted to segments of firing that were >10 spikes/sec to avoid fitting segments of the firing rate whether the afferent had been potentially driven into nonlinear cutoff:

1 $$\hat{{fr}}\left(t\right)=b+{S}_{v}\dot{H}(t)+{S}_{a}\ddot{H}(t)$$

where $\hat{{fr}}(t)$ is the estimated firing rate, ${S}_{v}$ and ${S}_{a}$ are coefficients representing the neuron’s modulation to head velocity and acceleration respectively, $b$ is a bias term representing the resting discharge. $\dot{H}\left(t\right)\,$is the head velocity in ^o^/s obtained from the gyroscope and $\ddot{H}(t)$is head acceleration in deg/s^2^ calculated from the derivative of the velocity. To ensure that there was little or no multicollinearity between our model inputs we used the variance inflation factor (VIF). In all cases, the VIF was lower than 1.2, strongly indicating the absence of multicollinearity. Additionally, we confirmed that residual values did not vary as a function of $\hat{{fr}}(t)$. The coefficients in Eq. 1 were then used to determine each neuron’s response modulation [S_t_ (sp/s)/(deg/s)] relative to head velocity using the following equation:

2 $${S}_{t}=\sqrt{[{{S}_{v}}^{2}+{\left(2\pi f{S}_{a}\right)}^{2}]}$$

A comparable approach was used to describe each otolith afferent’s response to translational head motion. Specifically, since the responses of otolith afferents are shown to lead to linear acceleration, we fit the following equation

3 $$\hat{{fr}}\left(t\right)=b+{S}_{a}\ddot{H}(t)+{S}_{j}\dddot{H}(t)$$

Where $\hat{{fr}}(t)$ is the estimated firing rate, ${S}_{a}$ and ${S}_{j}$ are coefficients representing the neuron’s modulation to head acceleration and jerk respectively, $b$ is a bias term representing the resting discharge, $\ddot{H}(t)$ is the head acceleration in g obtained from the accelerometer and $\ddot{H}\left(t\right)$ is head jerk in g/s calculated from the derivative of the acceleration. The coefficients in Eq. 3 were then used to determine each neuron’s response modulation relative to head acceleration^16^. As described above for the fitting of the linear model in Eq. 1, analysis was restricted to segments of firing that were >10 spikes/sec, and the assumptions required to model the time series using Eq. 3 were met.

4 $${S}_{t}=\sqrt{[{{S}_{a}}^{2}+{\left(2\pi f{S}_{j}\right)}^{2}]}$$

#### Response modulation and average firing rates

Prior studies of afferents during fictive locomotion (*larval xenopus*^13^) and preparation of an escape response (toadfish^11^) quantified vestibular afferents responses by computing mean firing rates and modulation sensitivities. The latter were calculated by taking the average peak-to-peak discharge modulation across cycles of motion stimulation. Accordingly, to facilitate direct comparison with these prior studies we used this same approach to compute the “modulation” of each afferent during walking and running. Additionally, we also computed the average firing rate of each afferent during walking and running to facilitate comparison with these prior studies.

#### Linear-nonlinear cascade models of vestibular afferents responses

To characterize our experimentally observed responses of vestibular afferents to locomotion stimuli, we used a linear-nonlinear (LN) cascade model in which the linear output firing rate $r(t)$ is given by the following:

5 $$r\left(t\right)={S}_{t}* H(t)+{r}_{0}$$

where ${S}_{t}$ is the neuron modulation and ${r}_{0}$ is the mean firing rate during passive stimulation. $H$ is the head motion (head rotational velocity for the canal afferents ($\dot{H}$) and head linear acceleration for the otolith afferents ($\ddot{H}$)). For modeling the neuron’s nonlinear response, actual firing rate and head motion signal (i.e, rotational velocity for the canal afferents and acceleration for the otolith afferents) were aligned by shifting the head motion signal by the values of the neuron’s lead found by using the cross-correlation (xcorr) MATLAB function. We then averaged the firing rate within 0.0004 g bins of the stimulus and computed the sigmoidal function that best fit the relationship between the neuron response and the linear prediction (Fig. 6 and Supplementary Fig. 4; see also ref. ^31^).

6 $${T}_{{sig}}\left(x\right)=\frac{c3}{2}\left[1+{erf}\left(\frac{x-c2}{\sqrt{2c1}}\right)\right]$$

in which $c1,c2\,,{{{{{\rm{and}}}}}}\,{c}3$, are fit parameters, and erf (·) is the error function. The neuron’s nonlinear response ($N(t)$) was then given by:

7 $$N\left(t\right)={T}_{{{{{{{\mathrm{sig}}}}}}}}({S}_{t}* H(t)+{r}_{0})$$

Response bias was taken as the firing rate at 0 head motion values and the neuron’s modulation was given by the slope of the sigmoid. For comparison, neuron response bias and modulation were reanalysed by using least-squares regression analysis (Eqs. 1 and 3) when the neuron was restricted to its linear range. The linear range was determined as the range of neuron responses around the mean value of the head stimulus ± one standard deviation (Fig. 4c). Similar bias and modulation values were found between the LN model and the least-squares regression analysis (all *p*-values > 0. 25).

### Quantification and statistical analysis

To evaluate the ability of the passive-based linear model or the nonlinear model to predict neuronal firing rates, we used Eqs. 1 and 3 to fit semicircular canal and otolith afferent responses, respectively, in the passive-pitch condition. We then used this linear model to predict the same afferent’s response during locomotion (walking and running) and computed the variance-accounted-for (VAF)^16^. For comparison, VAFs obtained using linear-nonlinear cascade models (Eq. 6) were also computed. The comparisons of the linear and nonlinear models for all classes of neuron for all conditions are depicted in Supplementary Figs. 5 and 6.

MATLAB (The MathWorks, Natick MA) and SPSS (IBM SPSS software, Armonk NY) was used for statistical analysis. Our sample sizes were similar to those generally employed in the field^17^. Before statistical analysis, the normality of distribution was evaluated using a Shapiro-Wilk’s test. All data were tested for the presence of non-stationarities using an augmented Dickey-Fuller test. We did not find any significant non-stationarities during either resting discharge or driven activities for any type of afferent (*p* > 0.05 in all cases). To determine if variances between groups were comparable, parametric tests were used. Statistical significance (*p* < 0.05) was determined using either two-tailed Student’s *t*-test or ANOVA when contrasting more than two means. Post-hoc pairwise comparisons were conducted using Dunn’s technique. Throughout, values are expressed as mean ± SEM.

### Reporting summary

Further information on research design is available in the Nature Research Reporting Summary linked to this article.

## Supplementary information

Supplementary Information

Reporting Summary

## Data Availability

The data analyzed and generating the resulting figures are provided with this paper and have been deposited in Github repository in the Source Data file: 10.5281/zenodo.5750946. Source data are provided with this paper.

## Source data

Source Data

## Code availability

Single-unit activity, gaze, head, and body signals and neural data were collected with the OmniPlex Neural Data Acquisition System (Plexon). After recordings, offline analyses of neural data were performed using custom algorithms (MATLAB, The MathWork). The MATLAB codes for nonlinear model are available through the link: 10.5281/zenodo.5750946.
