# Supplementary material for: Context-independent encoding of passive and active self-motion in vestibular afferent fibers during locomotion in primates
Source: Nat Commun. 2022 Jan 10;13:120. doi: 10.1038/s41467-021-27753-z (PMC8748921; doi:10.1038/s41467-021-27753-z)
Supplement: Supplementary file 1 — Supplementary Information [file 41467_2021_27753_MOESM1_ESM.pdf]

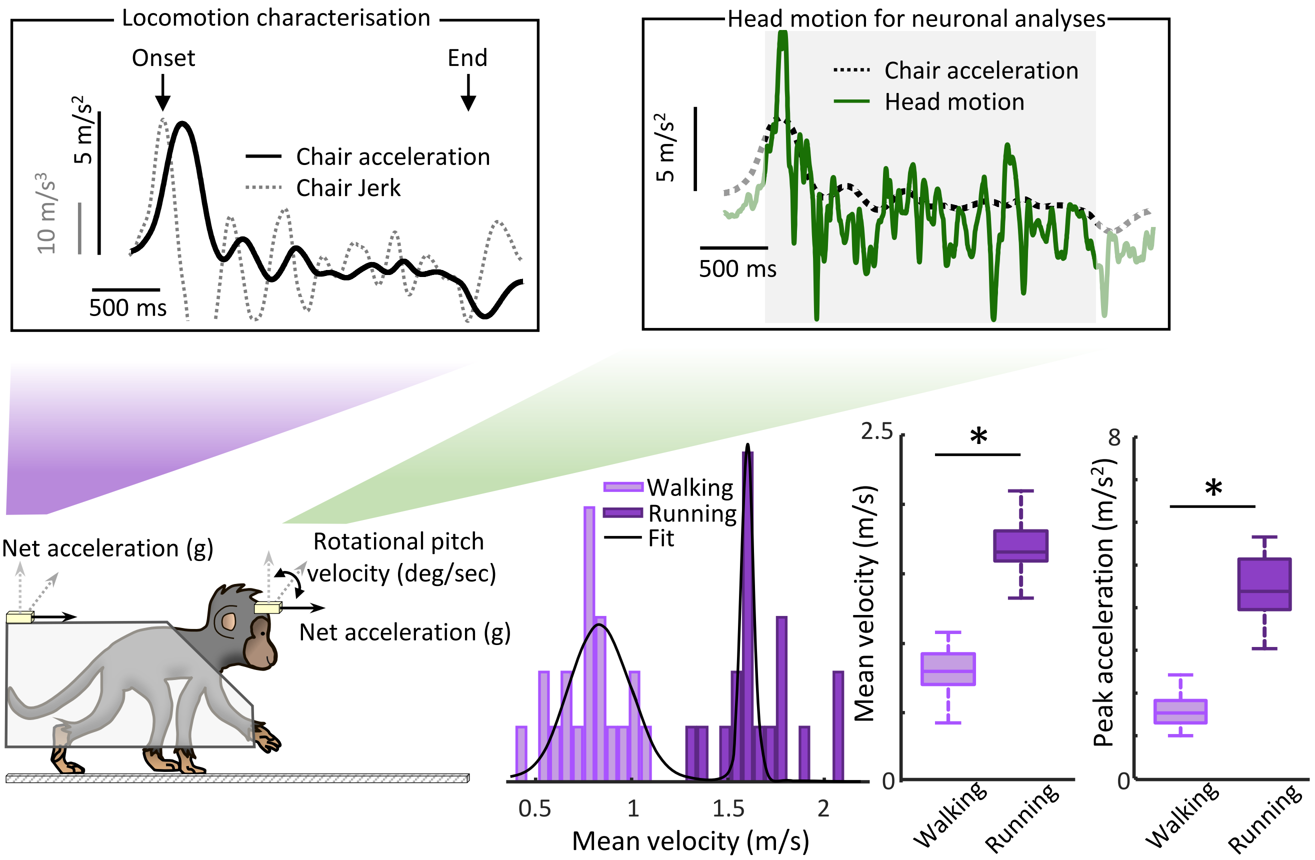

Supplementary Figure 1 : Patterns of locomotion. Once the bottom of the chair was removed, monkeys started walking or running through space along the 3.5-meter-long linear track. The characterization of the locomotor patterns was based on the motion sensor placed on the chair (purple shade, upper left inset) and head motion sensor was used for the neuronal analysis (green shade, upper right inset). The onset and offset of locomotion were defined as the last peak jerk preceding the peak acceleration or the peak deceleration of the body, respectively. The elapsed time between locomotion onset and offset was used to calculate the monkey's mean velocity (see onset and end arrows in upper left inset). The distribution of mean velocity was bimodal (Hartigan dip test, p = 0.01, central panel) and the mean velocities of locomotion segments classified as walking (light purple) fell within the first distribution while those classified as running (dark purple) fell within the second distribution. Mean velocity as well as peak acceleration were significantly different between walking and running segments (right most panels; n= 54, $t_{(54)}$ = 13.3, p = 1.5x10^{-8}, $t_{(54)}$, = -11.5, p = 3.7x10^{-9}, respectively). For all boxplots, the central mark indicates the median, the middle box indicates the 25th and 75th percentiles and the whiskers extend to the most extreme data points not considered outliers.

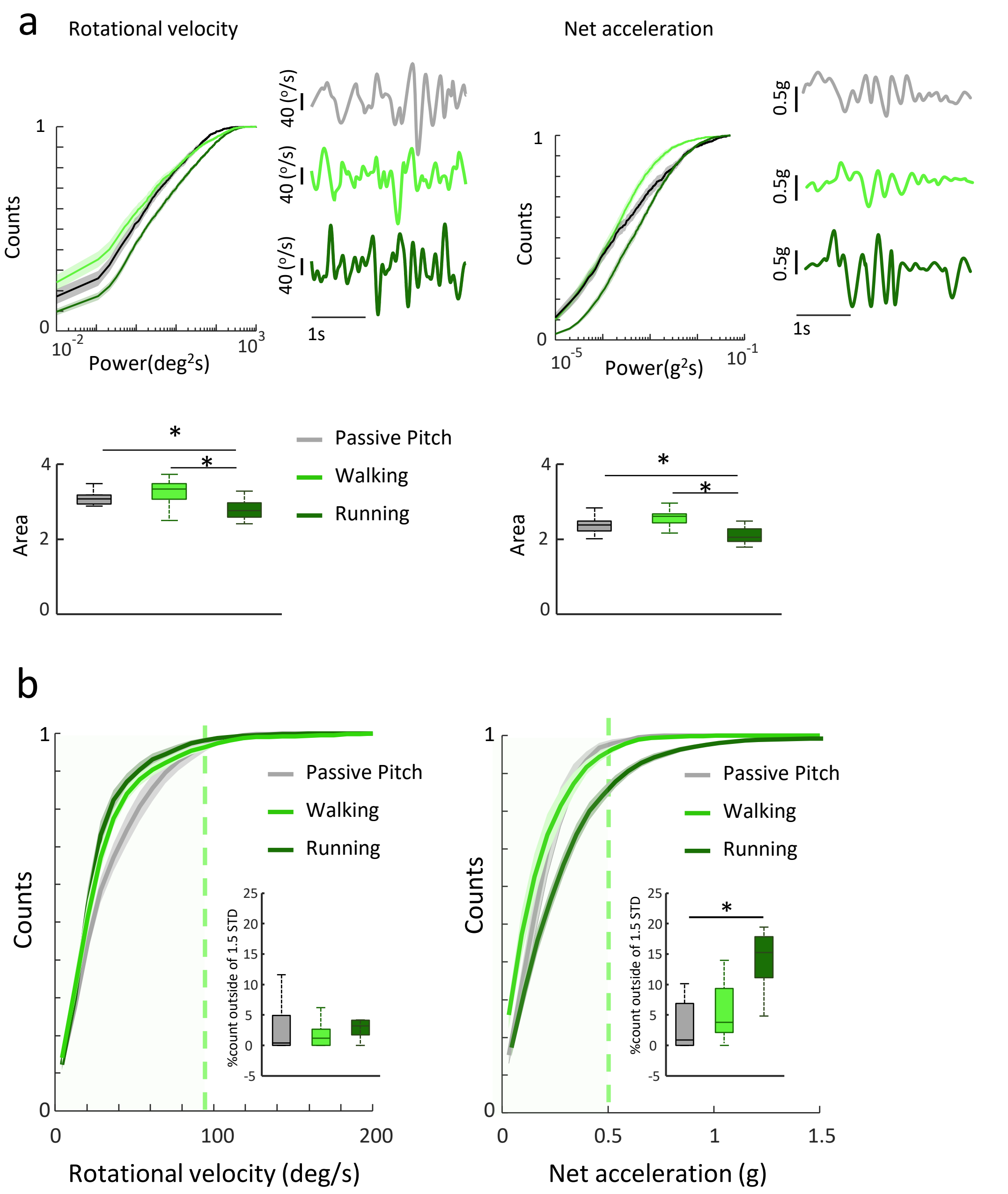

Supplementary Figure 2 : a) Cumulative histogram of the spectral power shown in figure 4 for the head velocity (left panel) and the net acceleration (right panel). The area under the curve was greater for passive pitch than walking than running for both rotational velocity (left bottom panel, n= 15, ANOVA $F_{(2,29)} = 11.6$, $p = 5.9\text{x}10^{-5}$) and net acceleration (right bottom panel, n= 11, $F_{(2,20)} = 17.91$, $p = 9.2\text{x}10^{-7}$). An example of rotational velocity time series as well as net acceleration time series are presented in the *insets*. **b)** Cumulative histogram of the absolute values for the head velocity (left panel) and the net acceleration (right panel). The percentage of the net acceleration that is outside of the linear range during running is larger than for passive pitch and walking (right bottom panel, $\text{Friedman}_{(2,20)} = 18.8$, $p = 8.5\ \text{x}10^{-5}$) which was not the case for the rotational velocity (left bottom panel, $\text{Friedman}_{(2,29)} = 4.0$, $p = 0.13$). For all boxplots, the central mark indicates the median, the middle box indicates the 25th and 75th percentiles and the whiskers extend to the most extreme data points not considered outliers.

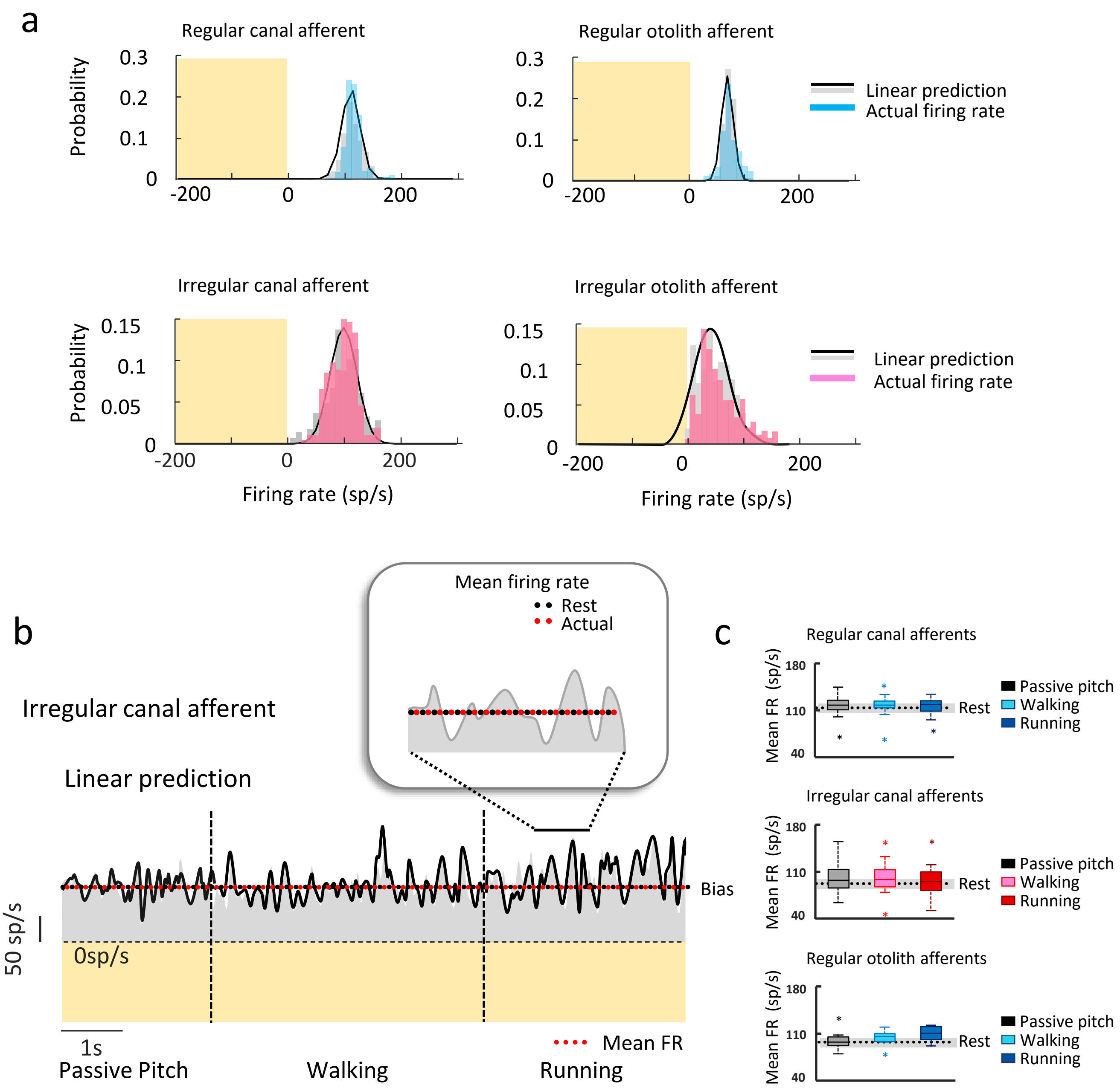

Supplementary Figure 3 : a) Probability distributions of the actual firing rates (blue, red) recorded from our example afferents during walking compared to the probability distributions linear model predictions (gray). **b)** The linear prediction model provided a good fit to the actual firing rate recorded from the example irregular semicircular canal during passive pitch, walking and running. *Inset:* Modulation of the example irregular semicircular canal afferent's response was symmetric around its resting discharge. Accordingly, mean firing rate and bias show identical values. **c)** Population-averaged mean firing rates and biases estimated using linear model predictions were comparable for regular semicircular canal afferents (n = 15, ANOVA, $F_{(3,42)} = 0.87$, $p = 0.46$), for irregular semicircular canal afferents (n = 17, ANOVA, $F_{(3,48)} = 0.96$, $p = 0.42$) or for regular otolith afferents (n = 9, ANOVA, $F_{(3,24)} = 2.71$, $p = 0.07$). For all boxplots, the central mark indicates the median, the middle box indicates the 25th and 75th percentiles and the whiskers extend to the most extreme data points not considered outliers.

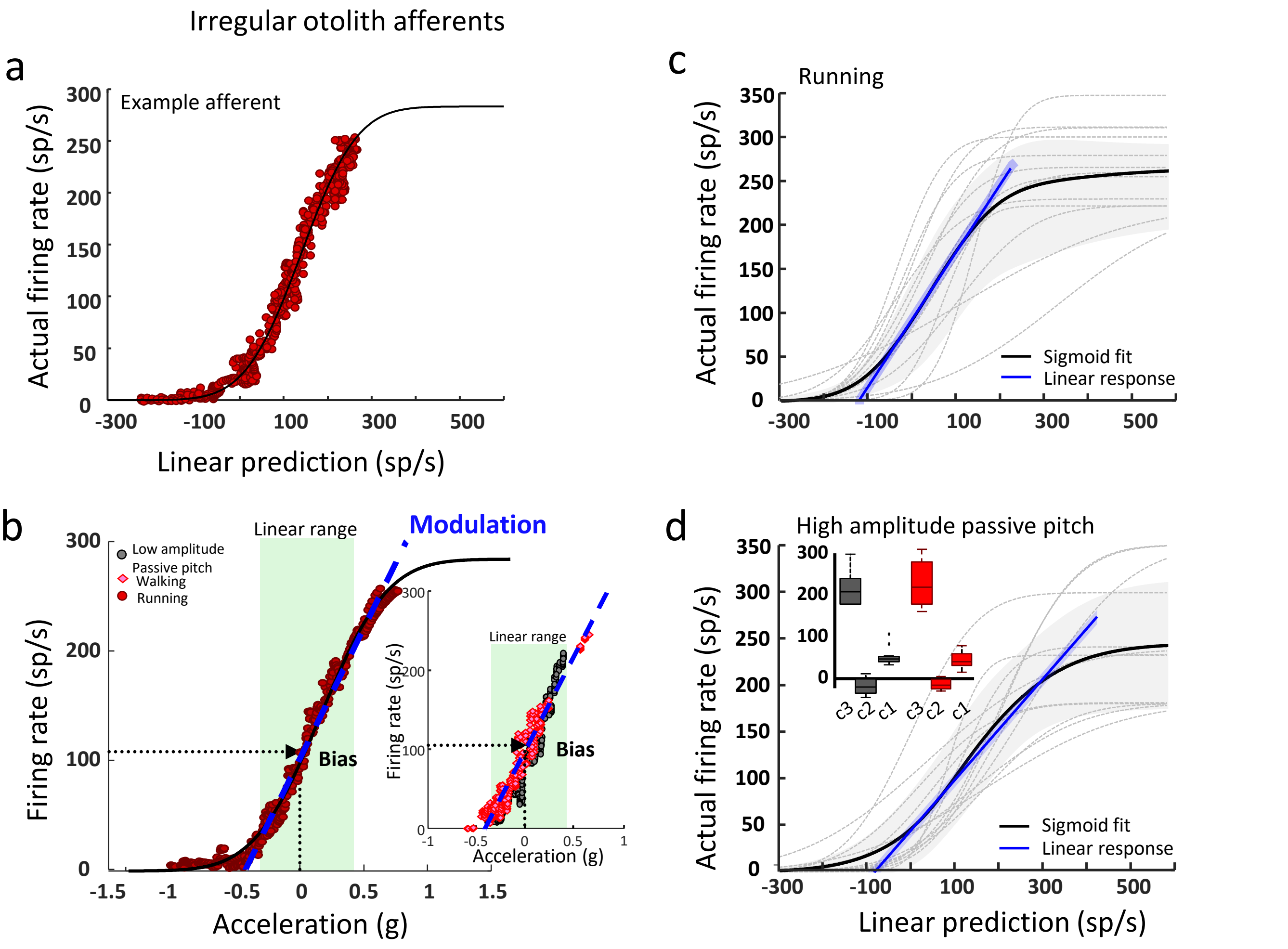

Supplementary Figure 4 : a) The relationship between the example irregular otolith afferent's response during running and the linear prediction is a nonlinear function that is well fit by a sigmoid (black line, VAF = 0.97). **b)** In its linear coding range (green box, Fig. 4c), the example irregular otolith afferent's response during running was similar to its response during low-amplitude passive stimulation and walking. The dashed black arrows show the value of the firing rate at 0g acceleration, which corresponds to the resting bias in *Equation 3*. **c)** The relationship between each individual irregular otolith afferent's response during running and the linear prediction was well fit by a sigmoid (mean VAF = 0.96 ± 0.01). **d)** Likewise, the relationship between each individual irregular otolith afferent's response during high-amplitude passive stimulation and the linear prediction was well fit by a sigmoid (mean VAF = 0.93 ± 0.04). *Inset:* The sigmoid coefficients that best fitted the relationship of each individual irregular otolith afferent's response to its linear prediction did not differ between running and high-amplitude passive stimulation (n = 14, Student t-test, all *ps*> 0.34). In both panels, the solid black sigmoid represents the population average and the shaded gray region represents standard deviation. The superimposed blue line represents the slope of the population-averaged sigmoid (i.e., population-averaged modulation in the linear regime), and the shaded blue region represents standard deviation. For all boxplots, the central mark indicates the median, the middle box indicates the 25th and 75th percentiles and the whiskers extend to the most extreme data points not considered outliers.

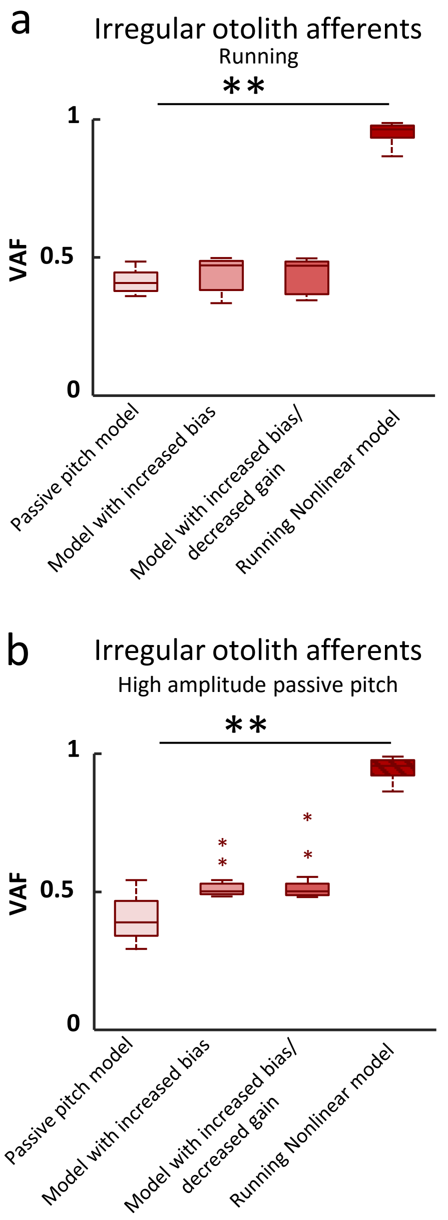

Supplementary Figure 5 : Comparison of the goodness of the fit for the linear and the nonlinear models to irregular otolith afferent responses during running (n = 14). Three optimized linear models (*Equation 3*) based on passive-based linear model responses for which i) the resting bias and modulation was held constant, ii) the resting bias was increased, and the modulation was held constant, and iii) the resting bias was increased, and modulation was decreased were tested for the high amplitude passive pitch and the running conditions. **a)** The nonlinear model provided a significantly better fit to the data than the linear models during running (ANOVA, $F_{(3,36)} = 723.8$, $p = 3.23 \times 10^{-32}$). **b)** Similarly, high amplitude passive pitch shows a significant increase in VAF for the nonlinear model compared to all linear models tested (ANOVA, $F_{(3,36)} = 34.4$, $p = 1.15 \times 10^{-10}$). Fitting the running nonlinear model on the high amplitude passive pitch data provided a better fit than the linear models (ANOVA, $F_{(3,36)} = 34.4$, $p = 1.15 \times 10^{-10}$). Finally, linear model did not differ from each other (all p = values > 0.92). For all boxplots, the central mark indicates the median, the middle box indicates the 25th and 75th percentiles and the whiskers extend to the most extreme data points not considered outliers.

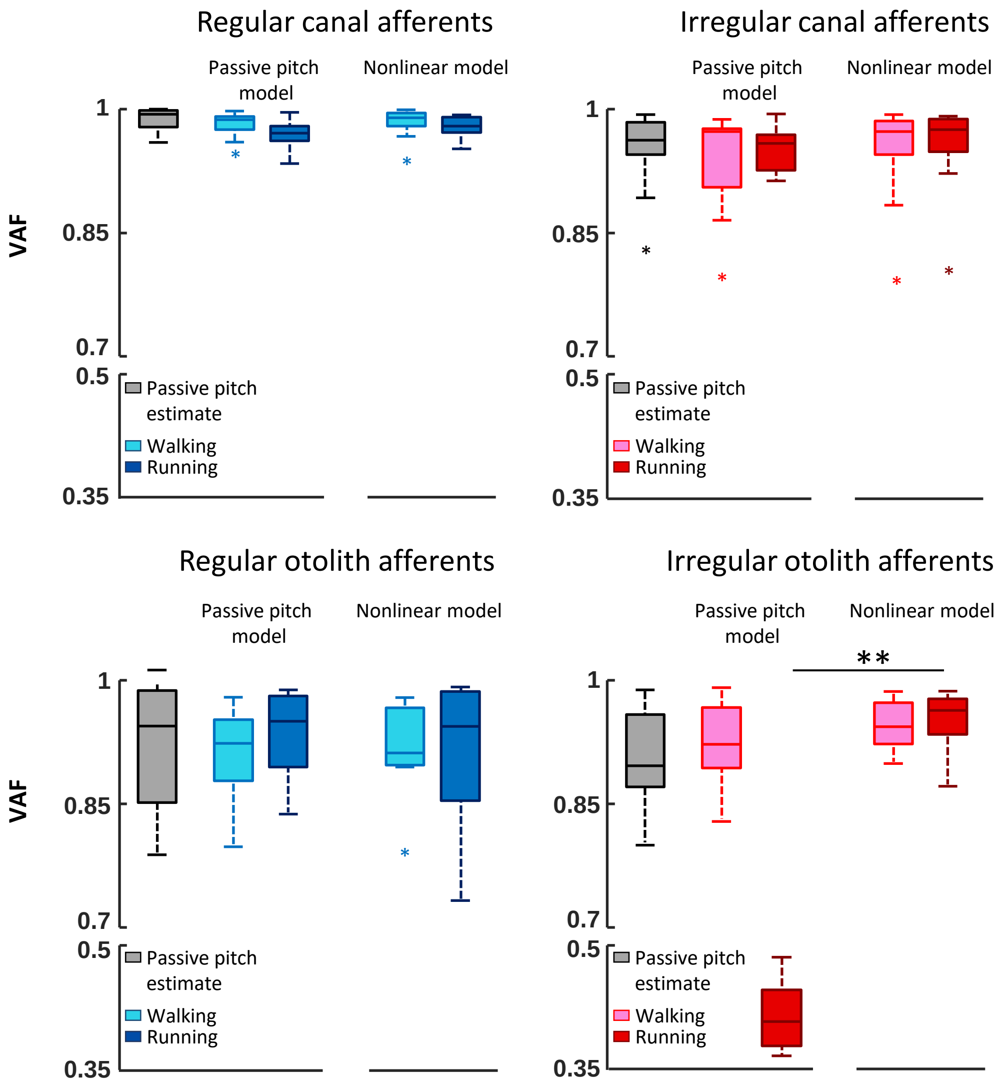

Supplementary Figure 6 : Comparison of goodness-of-fit (VAF) of the passive-based linear model and nonlinear model during walking and running for all afferents classes. *Top panels:* For canal afferents, the passive-based model well fitted the neuron response during walking and running. Nonlinear model did not provide a better fit to the neuron response, (upper panels: ANOVA, $F_{(4,56)}$ = 2.1, p = 0.41, $F_{(4,64)}$ = 0.73, p = 0.58, for the regular (n= 15) and irregular canal afferents (n =17), respectively). *Bottom panels:* For the regular otolith afferents (n =9), nonlinear model did not provide a better fit to the neuron response, $F_{(4,32)}$ = 1.33, p = 0.28. Conversely to that observed during the high amplitude passive pitch and the running condition, nonlinear model did not provide a better fit to the irregular otolith afferents response during walking (p = 0.99). For all boxplots, the central mark indicates the median, the middle box indicates the 25th and 75th percentiles and the whiskers extend to the most extreme data points not considered outliers.

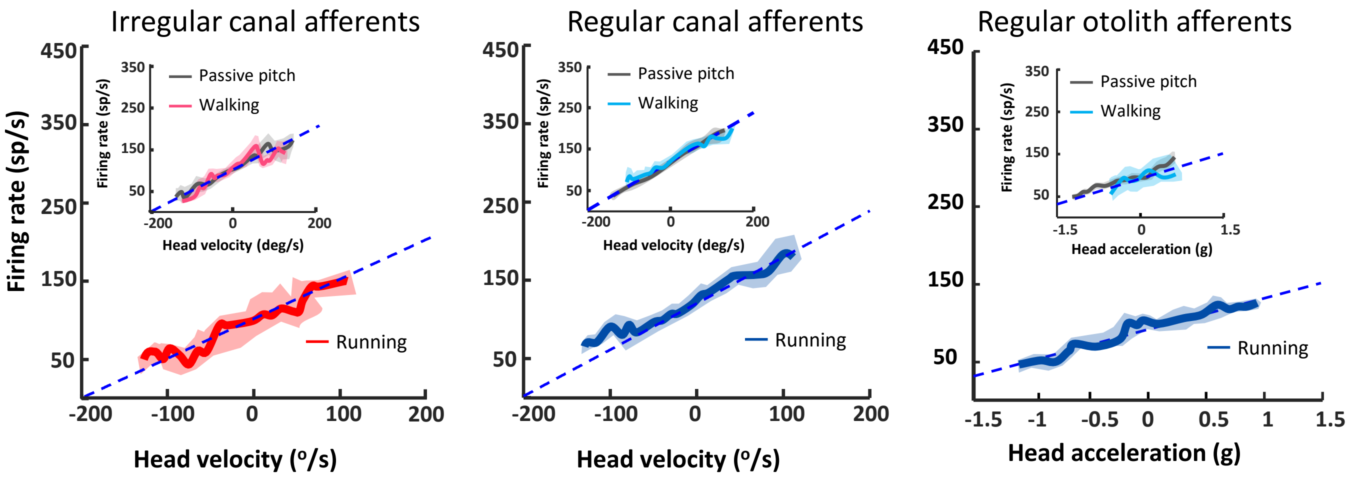

Supplementary Figure 7 : Example irregular (*left panel*) and regular (*right panel*) canal afferents (n =14 and n = 17) as well as example regular otolith (n =9, *right panel*) afferent responded linearly during naturalistic pitch, walking (inset), and running (mean ± STD).
