# Supplementary material for: Context-independent encoding of passive and active self-motion in vestibular afferent fibers during locomotion in primates
Source: Nat Commun. 2022 Jan 10;13:120. doi: 10.1038/s41467-021-27753-z (PMC8748921; doi:10.1038/s41467-021-27753-z)
Supplement: Supplementary file 2 — Reporting Summary [file 41467_2021_27753_MOESM2_ESM.pdf]

nature portfolio

Corresponding author(s): Kathleen E. Cullen

Last updated by author(s): Nov 9, 2021

Reporting Summary

Nature Portfolio wishes to improve the reproducibility of the work that we publish. This form provides structure for consistency and transparency in reporting. For further information on Nature Portfolio policies, see our Editorial Policies and the Editorial Policy Checklist.

Please do not complete any field with "not applicable" or n/a. Refer to the help text for what text to use if an item is not relevant to your study.
For final submission: please carefully check your responses for accuracy; you will not be able to make changes later.

Statistics

For all statistical analyses, confirm that the following items are present in the figure legend, table legend, main text, or Methods section.

n/a	Confirmed	
☐	☒	The exact sample size (*n*) for each experimental group/condition, given as a discrete number and unit of measurement
☐	☒	A statement on whether measurements were taken from distinct samples or whether the same sample was measured repeatedly
☐	☒	The statistical test(s) used AND whether they are one- or two-sided<br>*Only common tests should be described solely by name; describe more complex techniques in the Methods section.*
☒	☐	A description of all covariates tested
☐	☒	A description of any assumptions or corrections, such as tests of normality and adjustment for multiple comparisons
☐	☒	A full description of the statistical parameters including central tendency (e.g. means) or other basic estimates (e.g. regression coefficient) AND variation (e.g. standard deviation) or associated estimates of uncertainty (e.g. confidence intervals)
☐	☒	For null hypothesis testing, the test statistic (e.g. *F*, *t*, *r*) with confidence intervals, effect sizes, degrees of freedom and *P* value noted<br>*Give P values as exact values whenever suitable.*
☒	☐	For Bayesian analysis, information on the choice of priors and Markov chain Monte Carlo settings
☒	☐	For hierarchical and complex designs, identification of the appropriate level for tests and full reporting of outcomes
☒	☐	Estimates of effect sizes (e.g. Cohen's *d*, Pearson's *r*), indicating how they were calculated

Our web collection on statistics for biologists contains articles on many of the points above.

Software and code

Policy information about availability of computer code

Data collection	Single unit activity, gaze, body signals and neural data were collected with the OmniPlex Neural Data Acquisition System (Plexon)
Data analysis	After recordings, offline analyses of neural data were performed using custom algorithms (Matlab 2017, Mathwork)

For manuscripts utilizing custom algorithms or software that are central to the research but not yet described in published literature, software must be made available to editors and reviewers. We strongly encourage code deposition in a community repository (e.g. GitHub). See the Nature Portfolio guidelines for submitting code & software for further information.

Data

Policy information about availability of data

All manuscripts must include a data availability statement. This statement should provide the following information, where applicable:

- Accession codes, unique identifiers, or web links for publicly available datasets
- A description of any restrictions on data availability
- For clinical datasets or third party data, please ensure that the statement adheres to our policy

Data were deposited in FigShare: DOI: 10.6084/m9.figshare.16966654

Field-specific reporting

Please select the one below that is the best fit for your research. If you are not sure, read the appropriate sections before making your selection.

☒ Life sciences ☐ Behavioural & social sciences ☐ Ecological, evolutionary & environmental sciences

Life sciences study design

All studies must disclose on these points even when the disclosure is negative.

Sample size	Sample size comprise 55 neurons. The sample size were chosen based on previous studies that have recorded vestibular neurons. This sample size was shown to provide adequate statistical power.
Data exclusions	Neurons were excluded if isolation was lost before the end of the protocol.
Replication	All neurons were characterized using standard methods, and showed reproducibility with previous studies. The present experiment was performed independently for each neuron recorded and the results could be replicated at the sample size level.
Randomization	Randomization is not applicable as the neuron type is determined based on its physiology.
Blinding	Blinding is not relevant for our study because the neuron's response cannot be influenced by the experimenter.

Reporting for specific materials, systems and methods

We require information from authors about some types of materials, experimental systems and methods used in many studies. Here, indicate whether each material, system or method listed is relevant to your study. If you are not sure if a list item applies to your research, read the appropriate section before selecting a response.

Materials & experimental systems

n/a	Involved in the study	
☒	☐	Antibodies
☒	☐	Eukaryotic cell lines
☒	☐	Palaeontology and archaeology
☐	☒	Animals and other organisms
☒	☐	Human research participants
☒	☐	Clinical data
☒	☐	Dual use research of concern

Methods

n/a	Involved in the study	
☒	☐	ChIP-seq
☒	☐	Flow cytometry
☒	☐	MRI-based neuroimaging

Animals and other organisms

Policy information about studies involving animals; ARRIVE guidelines recommended for reporting animal research

Laboratory animals	The experiment was conducted in two male cynomologus monkeys (Macaca Fascicularis, 6 years old, 4 and 5kg, respectively).
Wild animals	The study did not involved wild animals.
Field-collected samples	The animals were housed in pairs on a 12 hour light/dark cycle.
Ethics oversight	All experimental protocols were approved by the McGill University Animal Care Committee (#2001-4096) and were in compliance with the guidelines of the Canadian Council on Animal Care.

Note that full information on the approval of the study protocol must also be provided in the manuscript.

This checklist template is licensed under a Creative Commons Attribution 4.0 International License, which permits use, sharing, adaptation, distribution and reproduction in any medium or format, as long as you give appropriate credit to the original author(s) and the source, provide a link to the Creative Commons license, and indicate if changes were made. The images or other third party material in this article are included in the article's Creative Commons license, unless indicated otherwise in a credit line to the material. If material is not included in the article's Creative Commons license and your intended use is not permitted by statutory regulation or exceeds the permitted use, you will need to obtain permission directly from the copyright holder. To view a copy of this license, visit http://creativecommons.org/licenses/by/4.0/
